# Comparative Evaluation of the Cavity Preparation Design on Mandibular First Molars in Typodont Teeth After Neurosculpting in Undergraduate Dental Students

**DOI:** 10.7759/cureus.86201

**Published:** 2025-06-17

**Authors:** Khushi A Desai, Khyati G Pandya, Ankit Arora, Sonali Kapoor, Harshil R Bhadang

**Affiliations:** 1 Department of Conservative Dentistry and Endodontics, Manubhai Patel Dental College and Hospital, Vadodara, IND

**Keywords:** dental education, dental training, dentist, dexterity, experimental study, fine motor control, neuroplasticity, psychomotor skills, undergraduate dental students

## Abstract

Introduction

Psychomotor skill development forms the cornerstone of competency in procedure-oriented professions, and dentistry is no exception. The dental practice is a complex interplay of neuroplastic abilities encompassing fine motor control, dexterity, visuospatial awareness, precise visuomotor coordination, finger dexterity, hand-arm steadiness, and multi-limb coordination for executing intricate procedures. Neuroplasticity is neuronal plasticity of the brain that occurs during learning motor skills. Hence, optimizing training methods becomes paramount. This study explores the potential of neurosculpting exercises (NSE) to enhance dental education by specifically targeting fine motor skills, dexterity, and neuromuscular coordination. The study aimed to evaluate the effects of the difference in class one cavity preparation design on typodont teeth done by undergraduate students after NSE.

Method

The study included all 40 students in the second-year Bachelor of Dental Surgery (BDS) undergraduate program. The students were randomly assigned to two groups (even and odd) of 20 each. In Group 1-experimental group (N = 20, 50%), students got regular pre-clinical practical training at the dental school and NSE for 15 minutes three times a day for two months. In Group 2-control group (N = 20, 50%), students received regular pre-clinical practical training at the dental school without NSE for two months. The class one cavity preparations of both groups were assessed and scored before and after intervention based on their outline form, depth, undercuts, and pulpal floor orientation parameters. The statistical analysis was done with the appropriate non-parametric tests: the Mann-Whitney U test for intergroup comparisons and the Wilcoxon signed-rank test for intragroup comparisons.

Results

The intergroup comparison of scores before the intervention between the control (N = 20, 50%) and experimental (N = 20, 50%) groups was negligible. The p-values were consistently above 0.05 (a p-value < 0.05 is significant), showing that both groups were comparable at baseline. At the end of eight weeks, both groups showed changes. However, the experimental group’s scores increased drastically when compared to the control group across all measures of the mean. The intragroup comparisons after intervention between the scores were statistically significant, as evidenced by p-values of 0.05 or less, highlighting the distinction in performance by the experimental group. This demonstrates a significant impact of the NSE intervention on the experimental group. The outline variable in the intragroup comparison of the experimental group improved from a mean of 5.36 (standard deviation (SD) = 1.07) before intervention to 6.22 (SD = 1.06) after intervention. Furthermore, the undercut scores improved from a mean of 5.14 (SD = 1.35) before the intervention to 6.67 (SD = 1.01) after the intervention.

Conclusion

NSE improved fine motor skills, leading to better results in complex tasks requiring precision. The p-values of significant variations suggestively explained the pivotal role of NSE in enhancing psychomotor skills. As the study proved positive, the NSE can be included in the curriculum, thereby evolving dental education.

## Introduction

Learning psychomotor skills necessitates the use of more creative teaching techniques, as these skills encompass both a cognitive/knowledge aspect and a motor aspect, unlike purely cognitive learning [1]. Mastering intricate psychomotor skills is a vital proficiency in dental training. The field of dentistry requires exceptional precision and expertise that necessitate the cultivation of cognitive development, unique dexterity, and motivation, all of which impact motor execution [2]. The progression from novice to specialist entails repeated practice and exposure to facilitate the formation of neuronal links in the brain. It will lead to the automation of the rehearsed movements [3]. This study investigates the potential benefits of combining neurosculpting with preclinical exercises for enhancing fine psychomotor skills. These findings aim to contribute to a growing understanding of how these techniques can work to improve mental agility and control. The brain's capacity for rewiring itself, called neuroplasticity [4], might be the advantage behind observational learning [5]. Since both involve forming new connections, this suggests that neuroplasticity could be critical for improving dexterity and fine motor skills just by watching others perform. Neurosculpting^®^ is the fusion of brain science and mindfulness, wherein Lisa Wimberger teaches few-minute exercises that trigger parts of the brain to activate the prefrontal cortex [6,7]. These exercises aim to improve focus, prefrontal brain activity, and resilience, all of which can strengthen psychomotor skills. They target different brain functions by coordinating physical movements with mental tasks. This neurosculpting exercise (NSE) was designed to develop visuospatial skills and hand-motor coordination relevant to cavity preparations. This is the first study to link neuroplasticity-inducing neurosculpting activities with dexterity skills in dentistry. This study is beneficial as it will help young dentists improve their dexterity abilities and aging dentists retain their dental expertise.

This study supports a fundamental revolution in dental education that prioritizes the development of outstanding psychomotor skills in future aspiring dentists. The aim is to create a standardized and skill-centric approach in the dental curriculum, thereby making practical expertise on par with theoretical knowledge, thereby enhancing the artistry part of dentistry.

## Materials and methods

This experimental type of study was conducted from August 11, 2023, to October 20, 2023, in the Department of Conservative Dentistry and Endodontics at Manubhai Patel Dental College and Hospital and Research Center in Vadodara, Gujarat, India. Following approval from the Institutional Ethics Committee (IEC) for research (IEC/MPDC_289/CONS-54/23), the study sought the proposed intervention of NSE.

Aims and objectives

The aim of this study is to evaluate the difference in class one cavity preparation design on typodont teeth done by undergraduate students after NSE. This study investigates the intriguing potential of NSEs to improve dental education. Through the development of students' fine motor skills and neuromuscular coordination, these exercises can help students become even more skilled clinicians. This research has the potential to make a huge impact on dental education. A favorable outcome could revolutionize the field and establish neurosculpting as the driving force behind the breakthrough. The objectives of this study are primarily to evaluate the cavity preparation design (the outline form, depth, undercut, and pulpal inclination using the Perfected for Amalgam Cavity Evaluation (PACE) instrument) of the control and experimental groups before the NSE intervention at week zero, as well as to evaluate the cavity preparation designs for the similar parameters of both the control and experimental groups after the NSE intervention at the end of week eight. Finally, another objective is to compare the scores of both the control and experimental groups (intergroup and intragroup comparisons). The sample size was established based on the number of second-year dental students available during the study period. The sample size represents the entire cohort of students who were available and willing to participate in the study. All 40 second-year Bachelor of Dental Surgery (BDS) dental students were enrolled in the research study. The inclusion criteria were a match for baseline data for class one cavity preparation design with variables of outline form, depth, undercut, and pulpal floor.

All the students (N = 40, 100%) did not have any neuromuscular abnormalities on examination. They were not on any medication or any drug dependency. All students provided written informed consent before the study. The even/odd approach was used to randomly divide all the students into two groups (N = 20, 50% for each group).

Group 1 (experimental group) consisted of 20 (N = 20, 50%) students with even-numbered roll numbers. They attended regular practical preclinical training at the dental school with three 15-minute NSE sessions per day.

**Figure 1:**
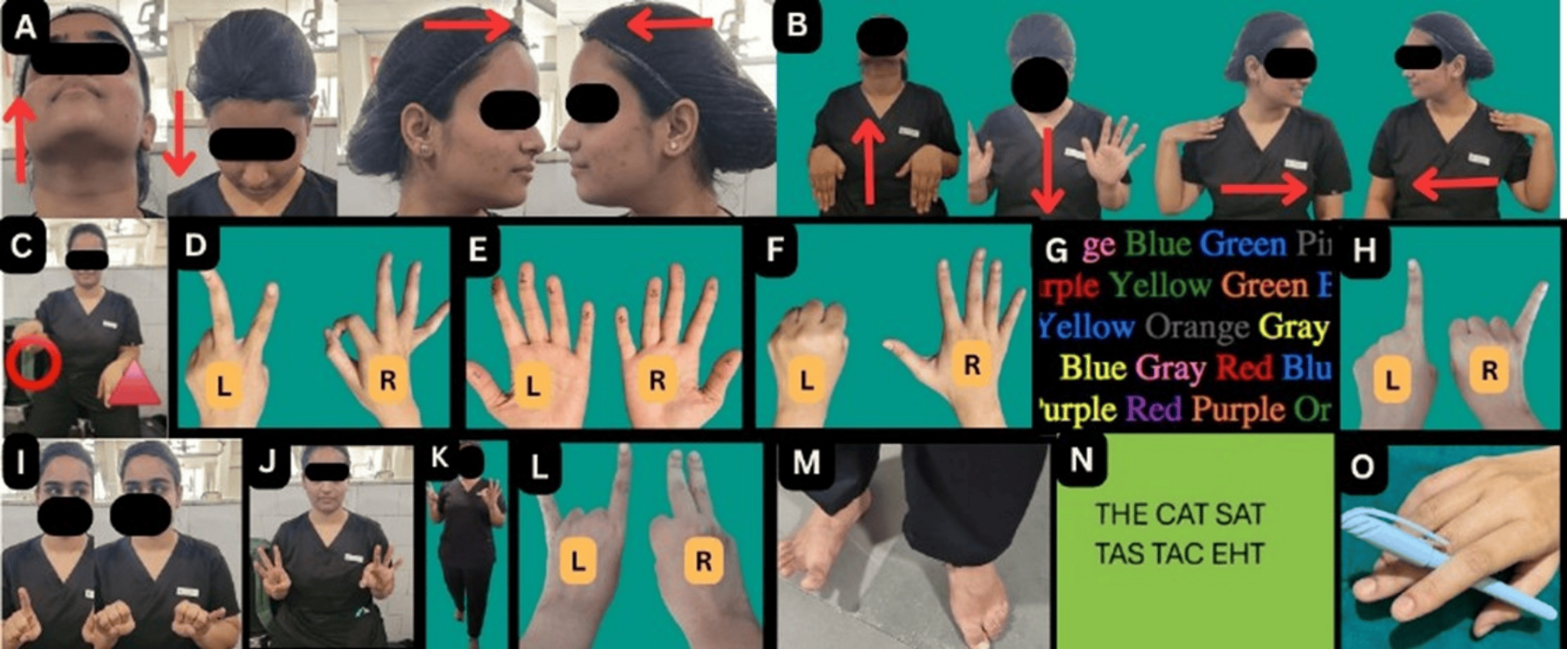
Picture collage illustrating the 15 neurosculpting exercises (NSEs) (A-O) performed by the experimental group Each picture of the collage demonstrates the head/hand/finger/eyes (I)/legs (M)/walk (K)/Stroop test (G)/reverse writing (N) movements in the respective NSE performed by the experimental group. These NSEs were performed for 15 minutes, three times a day for two months The arrows show the directions of head movements-up, down, left, and right L: left hand; R: right hand A: Exercise 1; B: Exercise 2; C: Exercise 3; D: Exercise 4; E: Exercise 5; F: Exercise 6; G: Exercise 7; H: Exercise 8; I: Exercise 9; J: Exercise 10; K: Exercise 11; L: Exercise 12; M: Exercise 13; N: Exercise 14; O: Exercise 15

The students followed the NSE schedule according to the level of difficulty. The NSE schedule followed a sequence that began with simple head-up-and-down exercises, then progressed to combining and synchronizing head movements with arm exercises, bimanual finger exercises, foot exercises, walking forward and backward doing hand exercises, and the gold standard Stroop test, culminating in the complete highest level of exercises of reverse handwriting and single-hand multiple-finger use by rolling a pen in between fingers (Figure 1). The technique of each exercise and the function that is strengthened are described below (Table 1). The experimental group of students performed these exercises with exquisite focus and proficiency. There was a defined schedule. The students began with Exercise No. 1 and continued it for four days (Day 1 to Day 4). A new NSE, Exercise 2, started on Day 5. Day 5's NSE schedule sequenced exercises from Day 4 and the new NSE for four days (Day 5 to Day 8). This pattern of exercises continued for 15 exercises (Exercises 1 to Exercise 15) up to 60 days (Table 2).

**Table 1:** Methodology of NSE and the brain function it enhances This table states the exercise number, name, performance technique, and the brain function it enhances. Letters (A) to (O) denote the picture demonstrations in Figure 1 of the respective exercises NSE: neurosculpting exercise

Name of exercise	Technique	Enhancer
Exercise 1: head exercise (A)	Move the head gently-up, down, left, and right	Builds focused attention
Exercise 2: arm-head exercises (B)	With both your arms raised in front of you (front raise) and the palms facing toward you and wrists down, slowly move your head up, then the head moves down, the palms facing away, wrists up. When the head moves to the right, the hand touches the left shoulder, and when the head moves to the left, the hand touches the right shoulder. Gradually increase speed	Focus enhancer
Exercise 3: circle-triangle exercise (C)	With both arms extended, draw a circle in the air with your right hand while simultaneously drawing a triangle with your left hand. Move in a clockwise direction and gradually increase speed (air drawing)	Brain engagement
Exercise 4: OK-peace hand sign exercise (D)	Make an "OK" sign with your right hand and a "peace" sign with your left hand. Quickly switch the signs between your hands, maintaining the opposite symbol in each hand. Increase speed as you become more comfortable	Mental challenge
Exercise 5: five-on-finger exercise (E)	Numbers 1 to 5 are randomly written on the fingers of both hands (in a different order). Starting from thumb to pinky finger, left hand: 1-4-2-3-5 and right hand: 4-1-2-5-3. The trainer calls out the numbers from 1 to 5 and 5 to 1. The students raise their fingers for the corresponding number	Increases focus and boosts neuroplasticity
Exercise 6: open-closed finger exercise (F)	The left hand forms a fist, while the right palm is open. The left hand will open fingers in the following order: pinky, ring, middle, index, and thumb. The right hand is open, and the fingers are closed one at a time in the following order: thumb, index, middle, ring, and pinky. Begin by softly moving one hand at a time, then both hands together. Gradually increase speed	Focused attention
Exercise 7: Stroop test (G)	Flashcards display a succession of words written in a variety of font colors. Students must vocally identify the hue of the font. They are not required to say the word, only the color of the font. The first round is slower, and the second is faster	Selective attention
Exercise 8: index pinky raise exercise (H)	Raise the right index finger while keeping the remaining fingers closed like a fist. Raise the left pinky finger while keeping the remaining fingers closed like a fist. Then, swap by raising the right pinky finger while keeping the remaining fingers closed like a fist, and the left index finger while keeping the remaining fingers closed like a fist. Begin with each hand slowly, one by one. Then both hands are brought together at the same time, and the speed is increased	Improves concentration
Exercise 9: finger-eye coordination exercise (I)	Do it without moving your head. The right index finger is raised, and the eyes look left. The left pinky finger is raised, and the eyes look to the right. Swap this finger pattern between the hands for 1 minute. The head should be kept straight and not moved. Do not look too far in one direction to avoid eye strain	Focused attention, stimulation of both sides of the brain, and builds brain resilience
Exercise 10: thumb-finger touch exercise (J)	Initiate the exercise using your right hand, bringing your thumb to touch the tip of each finger, starting with the index finger and progressing toward the pinky. Then perform the same action with your left hand, touching your thumb to the fingertips in reverse order, commencing with the pinky and concluding with the index finger. Begin using one hand at a time. Exercise with both hands simultaneously, gradually increasing the speed. Then, exercise with your palms facing downward	Develops focus and concentration
Exercise 11: thumb-finger step-up exercise (K)	Coordinate a walking-in-place exercise that includes alternating thumb-to-fingertip touches. Start by taking three steps forward while touching your right thumb to each fingertip in order (starting with the index and moving to the pinky) and simultaneously touching your left thumb to each fingertip in reverse order (starting with the pinky and moving to the index). After completing this, repeat the process while moving three steps backward	Increases complexity and brain challenge
Exercise 12: horn-finger exercise (L)	To form a "horn sign," extend your right hand's index and pinky fingers while extending the middle and ring fingers of your left hand. Start by practicing with each hand separately, then alternate the hand positions	Concentration booster
Exercise 13: foot-heel-toe exercise (M)	Point your right foot outward, heel down, toes up. Point your left foot outward, toes down and heel up. Start by practicing each foot movement separately. Next, practice both moves together and gradually increase the pace	Builds focus, resilience, and coordination
Exercise 14: reverse writing exercise (N)	Choose a simple sentence and write it down normally. Then, try writing the same sentence backwards, letter by letter. Simple sentence: "The cat sat." Backward: "tas tac ehT"	Mental agility
Exercise 15: pen rolling exercise (O)	Keep a pen between your thumb and index finger. Roll the pen around your palm, beginning with your index finger and progressing to each finger in order (index to pinky). Then, roll the pen back to your index finger	Dexterity and focus

**Table 2:** Set day-wise schedule for 15 NSE exercises (Day 1 to Day 60) The table states days (Day 1), old exercise (the day of the repeat exercise), and new exercise, which is added every fourth day For example, Day 1 (Exercise 1 conducted for four days) has no old exercise, and new exercise is Exercise 1. Day 5-Day 8 has to repeat the old exercises of Day 4 (Exercise 1) and the new learned exercise (of Day 5). In Exercise 2, the student performs Exercise 1 and Exercise 2 from Day 5-Day 8. The chart thereby continues as Day 1-Day 4, Day 5-Day 8 similarly up to Day 57-Day 60

Days	Old exercises	New exercises
Day 1-Day 4		Exercise 1
Day 5-Day 8	Day 4	Exercise 2
Day 9-Day 12	Day 8	Exercise 3
Day 13-Day 16	Day 12	Exercise 4
Day 17-Day 20	Day 16	Exercise 5
Day 21-Day 24	Day 20	Exercise 6
Day 25-Day 28	Day 24	Exercise 7
Day 29-Day 32	Day 28	Exercise 8
Day 33-Day 36	Day 32	Exercise 9
Day 37-Day 40	Day 36	Exercise 10
Day 41-Day 44	Day 40	Exercise 11
Day 45-Day 48	Day 44	Exercise 12
Day 49-Day 52	Day 48	Exercise 13
Day 53-Day 56	Day 52	Exercise 14
Day 57-Day 60	Day 56	Exercise 15

**Figure 2:**
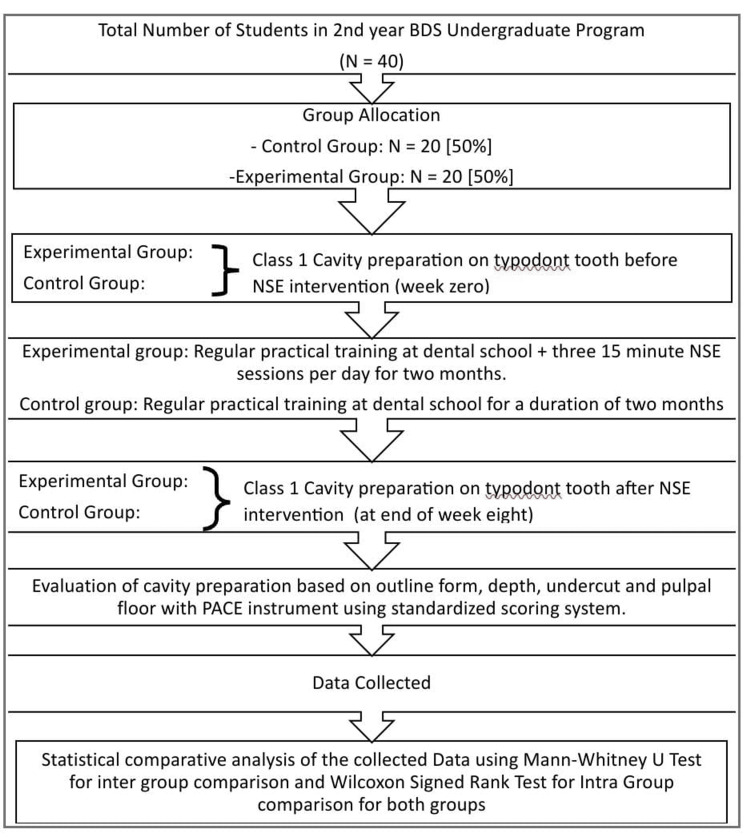
Flowchart illustrating the entire sequence of activities in the neurosculpting exercise (NSE) approach The figure gives a brief outline of the selection process, procedure steps, and analysis providing a comprehensive overview of how the NSE was implemented in the study BDS: Bachelor of Dental Surgery; PACE: Perfected for Amalgam Cavity Evaluation

Group 2 (the control group) had 20 (N = 20, 50%) students with odd-numbered roll numbers. They undertook regular preclinical practical training sessions at the dental school. These students did not participate in any NSE (Figure 2).

Assessment of cavity preparation

The study employed a single-blinded design. That is to say, the two examiners who evaluated the participants were unaware of whether the student belonged to the NSE intervention group or the control group (received regular practical training only). The reliability of the scoring process was assessed for interexaminer reliability.

In baseline assessment, all students participated in a standardized class one cavity preparation on the right mandibular molar on the typodont tooth at week zero. After the NSE intervention, both groups again participated in standardized class one cavity preparation at the end of week eight. Both assessments were based on outline form, depth, undercuts, and pulpal floor inclination. Students' cavity configuration skills were evaluated with the PACE instrument [8] and using a standardized scoring system (Table 3).

**Table 3:** Sample sheet of all the students’ class one cavity preparation scoring assessment form (before NSE/after NSE intervention) The scoring (standardized marking scheme) was based on the outline form, depth, undercut, and pulp floor orientation for each student of both groups NSE: neurosculpting exercise

	Outline form	Depth	Undercut	Pulpal floor orientation
Student 01				
Student 02				
Student 03				

After collecting the before and after intervention score data of both groups, data were assessed using non-parametric equivalents. The independent Student t-test or the Mann-Whitney U test was used for intergroup comparison, and the Wilcoxon signed-rank test was used to analyze intragroup differences. Statistical Package for the Social Sciences (SPSS) 20 software (IBM Corp., Armonk, NY, US) was used for the same.

The students were assessed based on the following marking scheme: 1-2: very poor; 3-4: poor; 5-6: acceptable; 7-8: very good; 9-10: excellent.

The intergroup and intragroup differences in parameters between control and experimental group students were compared using the independent Student t-test or the Mann-Whitney U test and the Wilcoxon signed-rank test depending on the distribution of data. SPSS 20 software (IBM Corp., Armonk, NY, US) was used for the same.

## Results

The intergroup comparison before the intervention using the Mann-Whitney U test results yielded the control group’s (N = 20, 50%) cavity preparation design's outline to pulpal mean of range 5.35 to 5.62 (standard deviation (SD) = 1.18 to 1.27) and experimental group’s (N = 20, 50%) mean of range 5.36 to 5.89 (SD = 1.07 to 1.31) (statistically significant p < 0.05). The p-values of range 0.976 to 0.4 were non-significant p-values (all p > 0.05), indicating there were no statistically significant differences between the groups for any of the measures before the intervention. This suggests that the groups were comparable at baseline data concerning outline form, depth, undercut, and pulpal before the start of the study. The overall skill for both groups was at par (Table 4).

**Table 4:** Intergroup comparison before the NSE intervention using the Mann-Whitney U test SD: standard deviation; NSE: neurosculpting exercise Data have been represented as cavity preparation variables (outline, depth, undercut, and pulpal), mean deviation, SD, Mann-Whitney U values, and p-value of both groups before the NSE intervention p-value is considered significant at p < 0.05

Before the NSE intervention: cavity preparation variables	Group				Mann-Whitney U	p-value
	Control N = 20 (50%)		Experimental N = 20 (50%)			
	Mean	SD	Mean	SD		
Outline	5.35	1.18	5.36	1.07	179	0.976
Depth	5.25	1.33	5.17	1.61	171	0.79
Undercut	5.50	1.53	5.14	1.35	146	0.318
Pulpal	5.62	1.27	5.89	1.31	152	0.4

The intergroup comparison of the cavity preparation parameters between the experimental and control groups after the intervention using the Mann-Whitney U test showed that the mean scores for all the variable range of 6.22 to 6.47 (SD = 1.06 to 1.08) and total score of 25.86 (SD = 3.86) were significantly higher in the experimental group than the control group whose mean scores were in the range of 4.98 to 5.17 (SD = 0.83 to 0.92) and total score of 20.47 (SD = 2.95). These indicate that the experimental group scored higher than the control group across all measures. This suggests that the NSE intervention had a substantial and statistically significant positive effect across all measured parameters. The p-values for all parameters were p < 0.001, indicating there was a significant statistical difference (statistically significant at p < 0.001) (Table 5).

**Table 5:** Intergroup comparisons after the NSE intervention using the Mann-Whitney U test SD: standard deviation; NSE: neurosculpting exercise *Significant value Data have been represented as cavity preparation variables (outline, depth, undercut, pulpal, and total), mean deviation, SD, Mann-Whitney U values, and p-value of both groups after NSE intervention p-value is considered significant at p < 0.05

After the NSE intervention: cavity preparation variables	Group					p-value
	Control N = 20 (50%)		Experimental N = 20 (50%)		Mann-Whitney U	
	Mean	SD	Mean	SD		
Outline	4.98	0.83	6.22	1.06	64.5	0.001*
Depth	5.23	0.90	6.50	1.10	67	0.001*
Undercut	5.10	1.01	6.67	1.01	52	<0.001*
Pulpal	5.17	0.92	6.47	1.08	66	0.001*
Total	20.47	2.95	25.86	3.86	44.5	<0.001*

The intragroup comparison shows that the changes in the control group both before and after intervention were generally mild, and none of these differences reached statistical significance as indicated by the p-values (all p > 0.05). In contrast, the experimental group showed statistically significant improvements after NSE intervention for outline, depth, undercut, and pulpal parameters, with p-values < 0.004, except for pulpal, which did not achieve significance. These results indicate that the intervention was effective in enhancing performance measures in the experimental group compared to the control group using the Wilcoxon signed-rank test (Table 6).

**Table 6:** Intragroup comparisons before and after NSE intervention using the Wilcoxon signed-rank test SD: standard deviation; NSE: neurosculpting exercise *Significant p-value Data have been represented as cavity preparation variables (outline, depth, undercut, pulpal, and total), mean deviation, SD, and p-value p-value is considered significant at p < 0.05

Group	Cavity preparation variables	Before NSE intervention		After NSE intervention		p-value
		Mean	SD	Mean	SD	
Control	Outline	5.35	1.18	4.98	0.83	0.113
	Depth	5.25	1.33	5.23	0.90	0.931
	Undercut	5.50	1.53	5.10	1.01	0.208
	Pulpal	5.62	1.27	5.17	0.92	0.121
	Total	21.73	4.41	20.47	2.95	0.09
Experimental	Outline	5.36	1.07	6.22	1.06	0.004*
	Depth	5.17	1.61	6.50	1.10	0.001*
	Undercut	5.14	1.35	6.67	1.01	0.002*
	Pulpal	5.89	1.31	6.47	1.08	0.152
	Total	21.56	4.36	25.86	3.86	0.001*

The mean difference intergroup comparison using the Mann-Whitney U test detailed the analysis. The lowest p-value among the cavity preparation variables was for outline and undercut: 0.001. The highest p-value of pulpal was 0.034, whereas the overall total p-value was <0.001, which was significant. The mean differences along with statistically significant p-values (p < 0.05) confirm that the NSE intervention led to substantial and significant improvements in the cavity preparation exercises done by the experimental group compared to the control group (Table 7).

**Table 7:** Intergroup comparison based on mean difference SD: standard deviation *Significant p-value Data have been represented as cavity preparation variables (outline, depth, undercut, pulpal, and total), mean deviation, SD, Mann-Whitney U values, and p-value p-value is considered significant at p < 0.05

Cavity preparation variables	Group				Mann-Whitney U	p-value
	Control (N = 20) (50%)		Experimental (N = 20) (50%)			
	Mean	SD	Mean	SD		
Outline	-0.38	1.04	0.86	1.01	71	0.001*
Depth	-0.02	1.55	1.33	1.36	90	0.008*
Undercut	-0.40	1.30	1.53	1.53	62	0.001*
Pulpal	-0.45	1.17	0.58	1.50	108.5	0.034*
Total	-1.25	3.09	4.31	4.35	46	<0.001*

## Discussion

Our study aimed to determine if combining NSE with preclinical training would enhance fine psychomotor skills and dexterity compared to preclinical training alone. We hypothesized that the combined approach would lead to a substantial improvement in mental agility and control during preclinical tasks. As expected, the group receiving NSE and regular preclinical dental training demonstrated a significant advantage in these skills compared to those who received only regular preclinical dental training. Researchers have proved through functional magnetic resonance imaging (fMRI) and repetitive transcranial magnetic stimulation (rTMS) that the areas of the brain involved in fine motor movements are the primary motor cortex, premotor cortex, presupplementary cortex, basal ganglia, supplementary cortex, posterior parietal cortex, and cerebellum [3]. The premotor complex mainly lies in the prefrontal area [9]. Neurosculpting is a process that involves triggering parts of our brain that we do not usually focus on. This activates the prefrontal cortex, which forms the basis for learning and mapping. It involves both hemispheres of the brain. When we combine mental activities with physical activities, it initiates the creation of new neural pathways [10]. Similarly, psychomotor skill learning is a combination of observation and practical learning, which is essential to refine a surgeon’s skills. Muscle memory and fine motor control develop through trial and error during procedures. This also creates new neural pathway connections in the brain, enabling more precise use of surgical instruments, thereby enhancing surgical skills [5]. fMRI shows different patterns of neural activity that occur in the prefrontal cortices, motor cortex, dorsal anterior cingulate gyrus, parietal gyrus, putamen, occipital gyrus, and cerebellum during motor activities. These new neural mechanisms engaged in motor performance and learning may inform NSE as novel interventions to enhance motor skill learning [11]. Dental schools give greater importance to theoretical knowledge, but our research suggests that there may be a potential training gap for developing important fine psychomotor practical skills. Second-year BDS students are a preferred group for participation in major educational research studies. Being early in their dental education (second year), these students have not formed ingrained habits yet. This allows researchers to assess the effectiveness of the training program without pre-existing biases. The evaluations by the examiners are less biased as it was a single-blinded study design. The intervention of NSE in our study included various essential parameters. Scientific research papers have shown that the schedule for NSE is based on repetitions, the number of sessions, and a stipulated time frame. Research reveals that mastering physical skills through brief bursts of practice interspersed with break periods is considerably more successful than pushing through lengthy sessions. Over time, NSE creates new brain pathways or connections. Some estimates place the NSEs’ duration at roughly eight weeks. Research has demonstrated that even very brief training regimens can have positive effects. Programs with 30 sessions, each lasting 10 minutes, can still be successful [4]. Scientists and physiologists draw on research and data that suggest the average attention span for effective learning is around 15 minutes. With this understanding, we have structured our exercises to last for 15 minutes, optimizing our learning potential during each session [12]. Movement science research indicates that strategically structuring work-rest cycles, with longer rest periods than work periods, enhances motor skill learning and performance [13]. Studies on skill acquisition further suggest that spaced practice, achieved through increased time between repetitions and sessions, improves fine motor skills [14]. Based on these findings, our training program incorporates three sessions of 15 minutes each. The findings suggest that rest periods should be longer than the actual work periods. A defined NSE schedule was followed according to the level of difficulty. NSE was structured as a sequential pattern/box pattern of learning. During learning trials, the researchers have found that blocked practice results in superior fine motor skill performance to random practice [15]. A study characterized stable motor patterns as similar to habits, which are defined as entrenched behaviors resistant to change through retraining [16]. This suggests that motor learning is a form of habit formation. Scientific evidence indicates that it takes a median of 59-66 days for individuals to reach peak automaticity in newly formed habits [17,18]. Finger exercises are done as "the brain allocates significant space to sensory information from the face and hand. Interestingly, the hand area has specific, non-overlapping regions for each finger" [19]. The experimental group’s class one cavity preparation parameters like outline, depth, and undercut showed improved scores after the NSE intervention. The pulpal floor showed no distinction, however. Following the intervention, there was a notable improvement in the overall score. There were only a few adjuncts that exhibited improvement. The standardized scoring system used to evaluate the students’ cavity preparation skills provided a clear and consistent method for examiners to score each student’s performance. The results indicated an improvement in psychomotor fine skills within the intervention group; the researchers planned to adhere to ethical principles by offering the same NSE training to the control group. Many efforts have been made to fill the void between undergraduate learning and the real world [20]. The present study is one step ahead and is aimed at working directly on the neural pathways.

A few methodological limitations must be acknowledged in our study. First and foremost, the study had a small sample size as the intake of the institute of one batch is 40 students for the second year. A future extension of this investigation to a larger cohort of subjects would generalize these findings. The adoption of the NSE technique as a part of dental curricular changes would demand a larger sample size. Furthermore, the study duration was only two months as motor learning and habit formation take a median of 59-66 days. A longer study duration would result in more evident neuroplastic changes in the brain thereby enhancing the precision of fine motor skills to a larger extent thereby measuring the long-term effects. Additionally, the surgical variant involved class one cavity preparation in a typodont tooth. The addition of other surgical variants would give more strength to the findings. The NSE was selected per recommendations by Lisa Wimberger [7]. However, multiple NSE other than the exercises in this study following the box sequential learning pattern would help in deeper neuroplasticity. Lastly, a subjective assessment of the effects of NSE and psychomotor skills was done. More objective assessment tools such as blood tests and neuroimaging tests-fMRI and rTMS-can directly measure the brain change and would be a stronger predictor of the neuroplastic changes in the brain.

## Conclusions

The experiment yielded a breakthrough: the experimental group achieved significantly better cavity parameters (outline, depth, and undercuts) while minimizing gross errors. NSE improved and enhanced fine motor skills, leading to better results in complex tasks that require precision. Integrating preclinical exercises with neurosculpting techniques may equip future dentists with exceptional surgical precision and mastery. This comprehensive approach could elevate the spectrum of dental skills, ultimately leading to a new generation of highly proficient clinicians. Students entering dental school could have excellent theoretical knowledge but would be lacking in fine motor skills. Therefore, these NSEs could be a boon to refine their dexterity, thereby comprehending their theoretical knowledge. Further research includes neuroplasticity to enhance psychomotor skills for remedial students with poor dexterity. Psychomotor skills and dexterity are vital to dentistry. The aging dentists lose their fine dental skills. Hence, neuroplasticity and aging dentists could be other research topics of interest. This research lays the groundwork for further exploration of methods for achieving flawless results and optimizing dental skills for the best possible outcomes with a commitment to excellence.
